# Student and faculty perceptions of, and experiences with, academic dishonesty at a medium-sized Canadian university

**DOI:** 10.1007/s40979-021-00090-w

**Published:** 2021-12-01

**Authors:** Oluwagbohunmi Awosoga, Christina M. Nord, Stephanie Varsanyi, Randall Barley, Jeff Meadows

**Affiliations:** 1 Faculty of Health Sciences (General), University of Lethbridge, 4401 University Drive West Markin Hall M3067, Lethbridge, AB T1K 3M4 Canada; 2 Faculty of Arts and Science, Department of Psychology, University of Lethbridge, Lethbridge, Alberta Canada; 3 Faculty of Arts and Science, Department of Biological Sciences, University of Lethbridge, Lethbridge, Alberta Canada; 4 Teaching Centre, University of Lethbridge, Lethbridge, Alberta Canada

**Keywords:** Academic dishonesty, Academic integrity, Academic misconduct, Canada, Student perceptions, Faculty perceptions, Policies, Academic disciplines

## Abstract

There is a paucity of research into the prevalence of academic dishonesty within Canada compared to other countries. Recently, there has been a call for a better understanding of the particular characteristics of educational integrity in Canada so that Canada can more meaningfully contribute to current discussions surrounding academic integrity. Here, we present findings from student (*N* = 1142) and faculty (*N* = 130) surveys conducted within a medium-sized (~ 8700 students) Canadian university. These surveys probed perceptions towards, and experiences with, academic dishonesty, in which we aimed to understand how students and faculty regarded academically dishonest practices during their postsecondary careers. We also aimed to understand how often students engaged in, and faculty had witnessed, academic dishonesty, whether or not witnessing incidents of academic dishonesty corresponded with gender, year of experience, highest level of educational attainment, discipline, or their personal perceptions towards the importance of academic honesty, and whether students had been adequately taught what constitutes academic dishonesty. We found that an overwhelming majority of students viewed academic honesty as important, and that most students reported not engaging in academic dishonesty themselves despite 45.8% reporting that they had witnessed others engage in academic dishonesty. We also found that students were more likely to witness cheating as their postsecondary experience increased, that witnessing varied across disciplines and educational attainment, and that witnessing varied with student perceptions. However, we found no such patterns in faculty responses, but found that faculty are split on whether or not they believe incidents of academic honesty are increasing.

## Introduction

We conducted an institutional self-study of student and faculty perceptions of, and experiences with academic dishonesty. We conducted this research for three main reasons. First, we wanted to understand whether our institution was experiencing an academic integrity crisis, as academic integrity has become a growing concern in colleges and universities. Our second reason was to add to the Canadian literature, which has experienced a paucity of research into academic integrity (Eaton and Edino 2018), particularly concerning contract cheating (Eaton et al. 2019; Lancaster 2019). Furthermore, exploring academic integrity at our medium-sized, teaching-focused institution also allowed us to investigate the relationship between academic integrity, university size, and faculty-to-student ratios, for which research is mixed (e.g., Arnold et al. 2007; Davis et al. 1992; McCabe et al. 1999; Tatum et al. 2018). Finally, we wanted to understand whether contract cheating, an increasingly growing concern for educators around the world, and particularly in Canada, where rates of contract cheating are largely unknown but have been previously reported as “all indications are that contract cheating is a problem” (Lancaster 2019, p. 8) was a problem at our university. This institutional self-study (Eaton et al. 2020) was a first step into in our efforts to use an evidence-based approach to institutionalize academic integrity as part of the university’s commitment to maintaining a tradition of academic integrity and personal civility within an environment that encourages intellectual exchange, creativity, originality, and discovery (Bertram Gallant and Drinan 2008; Eaton et al. 2020).

### Background

Academic dishonesty, cheating, and other forms of academic misconduct describe “a transgression against academic integrity, which entails taking an unfair advantage that results in a misrepresentation of a student’s ability and grasp of knowledge” (King et al. 2009, p. 4). In a recent review of the educational integrity research within Canada, it was found that most students are likely to engage in some form of academically dishonest practice during their careers (Peled et al. 2019; Stiles et al. 2017; Vandehey et al. 2007).

Of particular concern recently is the rise in contract cheating, defined as work submitted to educators by students who present it as their own work when, in fact, the work was completed by a third party. We prefer this “inclusive” definition (Eaton et al. 2019) because it is useful for investigations interested in the teaching and learning perspective of contract cheating and emphasizes that the student has actively opted out of the learning process, rather than requiring some monetary transaction to have occurred, as in some definitions that are more-so interested in the market components of contract cheating (e.g., students use of businesses such as assignment completing services, Rigby et al. 2015). The rates of contract cheating in Canada are largely unknown, but, using previous work and data from Statistics Canada, Eaton et al. (2019) estimated that up to 71,000 postsecondary Canadian students have engaged in contract cheating.

However, more work needs to be done within Canada. Eaton and Edino (2018) found that in the 25 years prior to 2018, studies concerning academic dishonesty/integrity conducted within Canada were somewhat split across descriptive/qualitative studies versus analytical/quantitative studies (54.4% versus 44.6%, respectively). Eaton and Edino (2018) also found that these studies mostly focused on students, most of which was quantitative in nature. However, the authors identified only one such study focused on faculty, and only found a handful of papers focused on both students and faculty. Eaton and Edino (2018, pp. 17-18) concluded that research contributions from Canada concerning academic integrity are “notably impoverished,” and called for “an increase in evidence-based, investigator-led, and funded research to better understand the particular characteristics of educational integrity in Canada and more intense participation in the ongoing global dialogue about integrity.”

The University of Lethbridge, located in Southern Alberta and founded in 1967 on traditional Blackfoot land, bills itself as “Alberta’s Destination University” and prides itself on small student-faculty ratios and ability to foster relationships between students and faculty. The University of Lethbridge has always emphasized undergraduate teaching, and, over the past 10 years, the University of Lethbridge has specifically sought to balance research priorities with a focus on the impact and importance of teaching (University of Lethbridge 2018). Through key appointments of senior positions, the institution has sought to elevate the value of the teaching that takes place without sacrificing the quality and importance of the research that helps inform that teaching. We have also experienced relatively low rates of academic offenses officially reported by faculty, with rates representing, on average, one half of 1 % of the student body. Because our university has emphasized the relationship between faculty and students, and academic dishonesty has been suggested to occur less often when there is a trusting relationship between educators and students (Morris and Carroll 2015), we were curious if our university’s efforts to emphasize a relationship between faculty and students has resulted in a low prevalence of academic dishonesty. However, the extent to which students were engaging in academic dishonesty that either went undetected or was not officially reported by faculty to the dean was largely unknown, and thus we lacked key data in understanding the efficacy of the university’s efforts to support teaching in general, and academic integrity in particular.

Furthermore, our university has no institutional-wide academic policy, with policies spread across the Principles of Student Citizenship, an undergraduate policy, and a graduate policy. There is no formal training in which students are taught to become familiar university polices regarding academic integrity, and consequences are largely up to the discretion of the faculty, and can range from the student needing to complete additional work, to expulsion. We believe this places our university somewhere between stage 1, “primitive, minimal policies and procedures” and 2, “radar screen, a set of policies and procedures in place but not fully developed or followed” of Pavela’s four categories of academic dishonesty policies (Pavela 1997). Crafting thoughtful and carefully developed polices have been shown to be important in developing a culture of academic integrity (Morris and Carroll 2015), and good academic integrity policy can reduce academic dishonesty and increase academic integrity (MacLeod and Eaton 2020).

Thus, concern for the rise in academic dishonesty, and contract cheating in particular, alongside interest in understanding the extent to which institutional interventions not directly aimed at academic integrity has nonetheless impacted it, led us to conduct a study at our own institution to ascertain if academic dishonesty is an issue at our medium-sized (~ 8700 students), mostly undergraduate Canadian university. By considering both student and faculty perceptions of, and engagement with, academic dishonesty at our institution, we hoped to not only understand the particular features and needs regarding academic integrity at our own institution, but to also add to the literature concerning academic integrity in Canada, and ultimately assist in the development of a common understanding of academic integrity institutionalization challenges (Bertram Gallant and Drinan 2008).

### Conceptual approach

Bertram Gallant and Drinan (2008) developed a model of academic integrity institutionalization. Academic integrity institutionalization is a term used by Bertram Gallant and Drinan (2008) to refer to the application of institutional theory in the establishment of academic interiority whereby factors that influence behaviors and inhibit or stimulate institutionalization are considered at the institutional, rather than individual, level. Thus, rather than consider academic dishonesty a problem comprised by the behaviors of the individuals involved, “[i]nstitutional theory suggests that an organization can mobilize around a change initiative or innovation, implement that innovation, and then see the innovation become stabilized or institutionalized within the organization” (Bertram Gallant and Drinan 2008).

Bertram Gallant and Drinan (2008) outlined four-stages of academic integrity institutionalization, with the first being “Recognition and Commitment.” Recognition and commitment entails recognition of the importance of academic integrity and voicing a commitment to it, and can include idea generation, evaluation, recognition of need, and the establishment of a need to respond to an issue. Institutional self-studies of academic dishonesty that are committed to using the results as part of an evidence-based effort to improve academic integrity is one clear avenue of recognition and commitment in academic integrity institutionalization (Bertram Gallant and Drinan 2008; Eaton et al. 2020).

In what follows, we present the results of our institutional self-study into the perceptions of, and engagement with, academic dishonesty. First, we explored the perceptions of and experiences with academic honesty at our university via a set of matching surveys given to students and faculty, with particular attention focused on incidents of contract cheating and self- plagiarism. After establishing that academic dishonesty does occur at the University of Lethbridge despite extremely low rates of contract cheating and self- plagiarism, we further interrogated our data to understand both why our university experienced such low rates of contract cheating, and where possible points of prevention and intervention would be most valuable for the kinds of academic dishonesty students did admit to engaging in. We first predicted that students who reported that they were adequately taught what constitutes academic dishonesty at our university would, as a consequence, witness incidents of academic dishonesty more than those who did not, as such students would be better able at identifying academic dishonesty in the first place. Given that longer tenure in postsecondary education increases the chances of interacting with others, we also expected that participants with more experience in postsecondary education would be more likely to report witnessing incidents of academic dishonesty and included experience to account for this possible effect. Additionally, we predicted that witnessing incidents of academic of dishonesty would also differ across discipline, given that different disciplines use different metrics and have different foci for student outcomes. Detecting such differences with regard to discipline is critical for developing evidence-based policies, and follows the recommendation of Eaton and Edino (2018) of including discipline-related analyses in academic integrity research. Finally, we also explored the extent to which student perceptions regarding academic integrity corresponded with whether or not they witnessed academic dishonesty.

## Methods

### Ethics approval

Human Subject Research Ethical Review and approval was obtained from the University of Lethbridge Human Subject Research Committee (Protocol #2019–084).

In October and November of 2019, we conducted two anonymous surveys concerning the perceptions of and experiences with academic dishonesty at the University of Lethbridge, a medium-sized university in, Southern Alberta, Canada. The University of Lethbridge’s main campus is located in Lethbridge, Alberta, approximately 100 km north of the Canadian/US border, and a satellite campus is located approximately 210 km north of Lethbridge in Calgary. In the Fall of 2019 when this study took place, 8795 (8155 undergraduate, 640 graduate) students were enrolled at the University of Lethbridge, 8.9% (785 students) of which were from out-of-province, and 10.8% of which were international students. The online surveys were administered using Qualtrics and sent to students and faculty across all faculties via email. The surveys included both similar and unique questions in order to address the unique perspectives of students and faculty. For example, while faculty were asked about their experiences with encountering academic dishonesty in their students’ work, students were asked whether they ever engaged in academic dishonesty. A full list of the questions asked on each survey are provided in the Appendix A.

### Participants

A total of 1142 students participated in the student survey, representing 12.9% of the student population at the university (Table 1). The majority of student participants were female (65.8%, only slightly higher than the 59% of all enrolled students) and between the ages of 20–29 (58.7%; average age of enrolled undergraduate students: 22). Student participants were fairly evenly spread across their first (22.3%), second (16.4%), third (20.5%), fourth (19.9%), and fifth (or more; 20.9%) year of study. Most students’ highest education attainment was a high school diploma (66.7%), and the majority of student participants were enrolled in either Arts & Science (Sciences, 34.8%; Social Sciences 13.6%), or Dhillon School of Business (15.5%).

**Table 1:** Student Participant Demographics

Demographic	N	Percentage
**Gender**		
Female	723	65.8%
Male	351	31.9%
Non-binary	14	1.3%
Do not wish to identify	11	1.0%
**Age**		
17–19	307	27.9%
20–29	646	58.7%
30–39	90	8.2%
40–49	40	3.6%
50–59	12	1.1%
60–69	1	0.1%
70 +	4	0.4%
**Year of postsecondary experience**		
^1st^ Year	245	22.3%
2nd Year	180	16.4%
^3rd^ Year	225	20.5%
^4th^ Year	219	19.9%
^5th^ or greater	230	20.9%
**Highest educational attainment**		
High school diploma	732	66.7%
Diploma or certificate	130	11.8%
Bachelor’s degree	187	17.0%
Master’s degree	34	3.1%
Doctorate degree	8	0.7%
Other	7	0.6%
**Discipline enrolled in**		
Arts & Science - Sciences	383	34.8%
Dhillon School of Business	171	15.5%
Arts & Science - Social Sciences	150	13.6%
Health Science/Nursing	104	9.5%
Fine Arts	91	8.3%
Arts & Science - Humanities	84	7.6%
Education	83	7.5%
Other	34	3.1%

For the faculty survey, a total of 130 participants participated, representing 22% of the faculty population at the university (Table 2). Faculty participants were almost evenly split across females (49.6%) and males (47.0%), and 78.1% were 40 years of age or older. The largest proportion of faculty had 11–20 years of postsecondary experience (33.9%), and 69.6% had a doctoral degree. Teaching positions were mostly split across associate professors (26.3%), professors (20.2%), assistant professors (14.0%), full-time academic assistants/instructors (19.3%), and sessional instructors (14.9%), with a minority of faculty participants representing those working term appointments (5.3%).

**Table 2:** Faculty Participant Demographics

Demographic	N	Percentage
**Gender**		
Female	58	49.6%
Male	55	47.0%
Non-binary	1	0.9%
Do not wish to identify	3	2.6%
**Age**		
20–29	6	5.3%
30–39	19	16.7%
40–49	40	35.1%
50–59	32	28.1%
60–69	14	12.3%
70 +	3	2.6%
**Postsecondary teaching experience**		
5 or fewer	24	20.9%
6–10	19	16.5%
11–20	39	33.9%
21–30	21	18.3%
31 or greater	12	10.4%
**Highest educational attainment**		
Professional designation	2	1.7%
Bachelor’s degree	8	7.0%
Master’s degree	25	21.7%
Doctoral degree	80	69.6%
**Discipline teaching in**		
Arts & Science - Sciences	35	30.2%
Dhillon School of Business	16	13.8%
Health Science/Nursing	15	12.9%
Arts & Science - Humanities	14	12.1%
Arts & Science - Social Sciences	12	10.3%
Fine Arts	10	8.6%
Education	8	6.9%
School of Liberal Education	2	1.7%
Other	4	3.4%
**Teaching position**		
Term Appointment	6	5.3%
Sessional Instructor	17	14.9%
Full-time Academic Assistant/Instructor	22	19.3%
Assistant Professor	16	14.0%
Associate Professor	30	26.3%
Professor	23	20.2%

### Descriptive and quantitative analysis

We conducted both descriptive and multivariate statistical analyses of the survey data in order to understand the perceptions towards, and experiences with, academic dishonesty of students and faculty at the university. We determined the range of student and faculty perceptions surrounding academic dishonesty, and self-reported engagement with academic dishonesty. Furthermore, we also conducted two binary logistic regressions using the Statistical Package for Social Sciences (SPSSInc 2017) to understand whether or not students witnessed incidents of academic dishonesty differed demographically, in accordance with their highest level of educational attainment, or with regard to their perceptions. While we also attempted to conduct these analyses for the corresponding questions in the faculty survey, these questions did not have enough variation for the assumptions of the logistic regression (i.e., observation must be independent, and predictors must be linearly related to the logit of the outcome variable; data received from faculty members violated this assumption hence we could not obtain 95% confidence interval for odds ratios of the predictors). Because of this, we have conducted a descriptive analysis of these questions in the same fashion as the logistic regressions for the student survey.

For our descriptive analyses, we produced tables and graphs of participant responses to our questions of interest. These questions included student and faculty perceptions towards academic dishonesty (Appendix A question 7, Appendix B questions 12 and 23), and self-reported participation in academic dishonesty (Appendix A questions 10–12; Table 3). Finally, because our sample lacked sufficient variation to conduct a regression analysis, we performed a descriptive analysis of faculty responses to the questions concerning their experiences of witnessing academic dishonesty and familiarity with university polies regarding academic dishonesty (Appendix B, questions 8 and 21) in accordance with the participant demographic of interest (Appendices D and E).

**Table 3:** Student Participation in Academic Dishonesty

Statement (n)	Yes (%)	No (%)
Have you ever reused an assignment for another course (at the post-secondary level)? (*n* = 893)	63 (7.1%)	830 (92.9%)
Have you ever turned in an assignment (at the post-secondary level) that someone else completed for you? (*n* = 894)	13 (1.5%)	881 (98.5%)
Have you ever turned in an assignment (at the post-secondary level) that you paid someone else to complete for you? (This does not include someone being paid to edit the paper for things like APA formatting, etc.)? (*n* = 893)	4 (0.4%)	889 (99.6%)

For our logistic analysis, we aimed to understand whether or not students who have witnessed incidents of academic dishonesty differed demographically, with their highest level of educational attainment, or in accordance with their perceptions. We set whether or not witnessed incidents of academic dishonesty in the past (no/yes, referred to as “n/y”) as our dependent variable, and gender, an interaction between year of postsecondary experience and highest level of educational attainment, discipline, perceptions regarding the importance of academic honesty among students, and perceptions regarding whether they had been adequately taught what constitutes constitutes plagiarism, academic fraud, academic misconduct or other cheating behaviours as the independent variables.

## Results

**Fig. 1:**
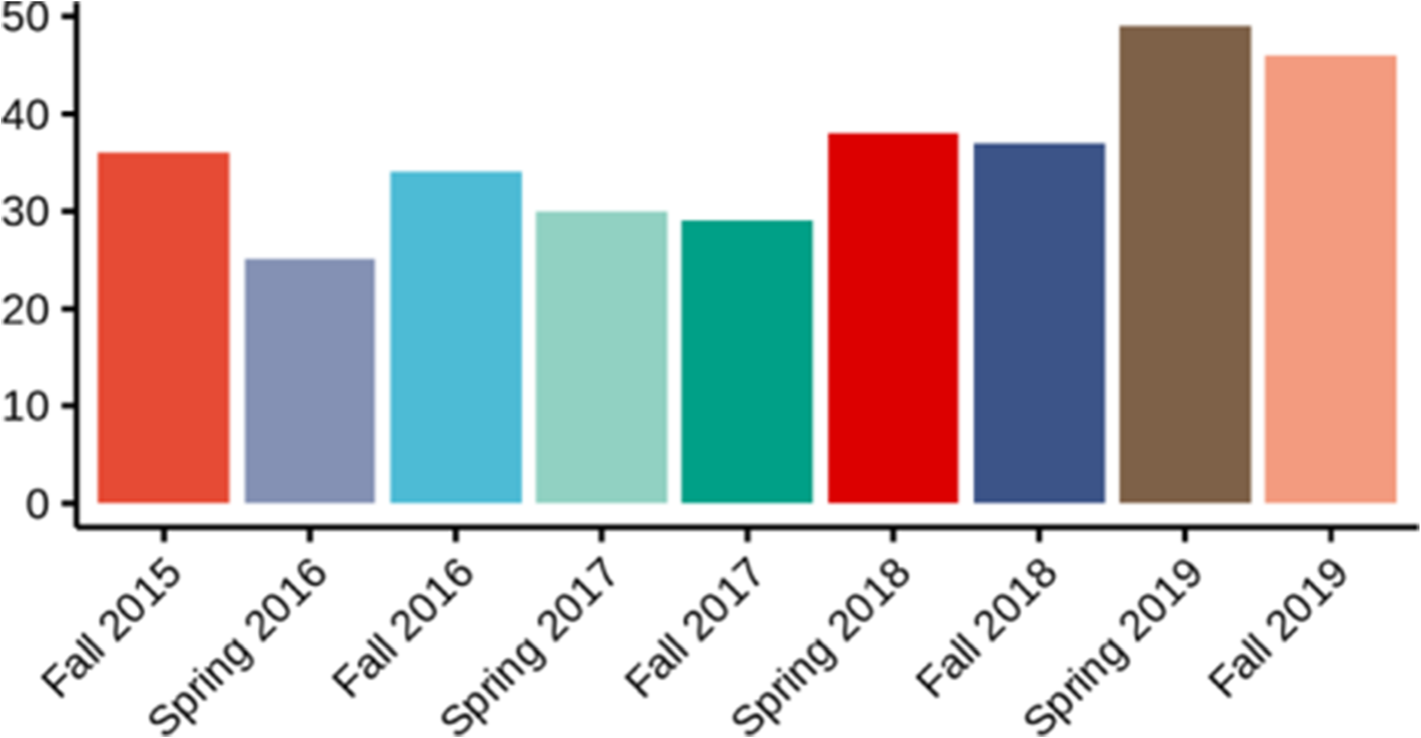
Student Academic Offenses By Semester

A total of 46 academic offenses by students were officially reported by faculty to the university during the time of our study (Fall 2019 semester), consistent with previous semesters (Fig. 1, Appendix C).

### Student perceptions

#### Students

**Fig. 2:**
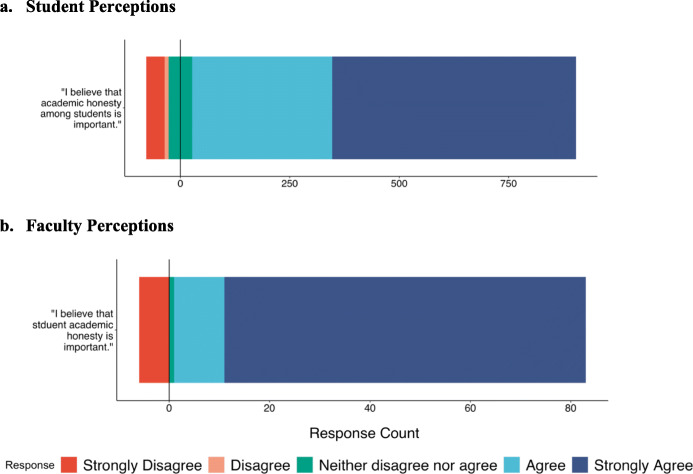
(**a**) Student and (**b**) Faculty Perceptions regarding Academic Honesty

Overall, 91.8% of student participants agreed that student academic honesty is important, 58.4% of which agreed strongly (Fig. 2a; Appendix D). Just 4.4% strongly disagreed with this statement, 0.9% disagreed, and 2.9% neither disagreed nor agreed. Over 83.0% of students agreed that they had been adequately taught what constitutes plagiarism, academic fraud, academic misconduct or other cheating behaviors at our university, and 33.3% agreed strongly; 2.0% strongly disagreed, 7.6% disagreed, and 8.5% neither disagreed nor agreed with this statement.

### Student participation in academic dishonesty

Only 7.1% of students answered “yes” to ever reusing an assignment for another course, while 92.9% answered “no” (Table 4). The majority of students answered that they had not asked another person to complete an assignment for them (98.5%), while 13 students, representing 1.5% of student participants, reported that they had. Finally, just 4 students, representing 0.4% of student participants, admitted to paying someone else to complete an assignment for them, while 99.6% answered that they had not done so (Table 4).

**Table 4:** Estimates of Whether Students Reported Witnessing any Incidents of Academic Dishonesty in the Past

Main Effect	β	SE	Sig.	Exp(β)	Lower 95% CI	Upper 95% CI
Intercept	−.678	.269	.012	.508		
**Males (Females)**	−.039	.170	.816	.961	.690	1.340
**Year of experience (1st year)**	.321	.112	.004	1.379	1.108	1.716
**Discipline (Arts & Sciences - Sciences)**			<.001			
Arts and Science - Humanities	−1.084	.310	<.001	.338	.184	.621
Arts and Science – Social Sciences	−.559	.245	.023	.572	.353	.924
Dhillon School of Business	−.057	.235	.810	.945	.596	1.499
Education	−.762	.307	.013	.467	.255	.852
Fine Arts	−1.144	.329	.001	.319	.167	.608
Health Sciences/Nursing	−.362	.278	.193	.696	.404	1.200
**Highest Level of Educational Attainment (High School Diploma)**			.171			
Diploma or Certificate	.282	.309	.361	1.326	.724	2.431
Bachelor’s degree	.862	.499	.084	2.368	.891	6.292
Master’s degree	1.853	.801	.021	6.378	1.327	30.656
Doctorate degree	2.524	1.42	.076	12.475	.772	201.678
**Belief that academic honesty among students is important (Strongly agree)**			.024			
Agree	−.337	.177	.057	.714	.505	1.009
Neither disagree nor agree	−1.099	.505	.030	.333	.124	.897
Disagree	2.051	1.130	.070	7.776	.848	71.289
Strongly disagree	−.236	.374	.528	.790	.380	1.643
**Belief that they have been adequately taught what constitutes constitutes plagiarism, academic fraud, academic misconduct or other cheating behaviours**			.040			
Agree	.278	.175	.113	1.321	.937	1.863
Neither disagree nor agree	.726	.314	.021	2.067	1.117	3.827
Disagree	.432	.311	.164	1.540	.838	2.832
Strongly disagree	1.305	.574	.023	3.688	1.196	11.369
**Interaction of education and experience**	−.085	.061	.167	.919	.815	1.036

Gender is relative to females, discipline is relative to Arts & Sciences – Sciences, highest level of education is relative to High School Diploma, and all Likert responses are relative to Strongly Agree. The model correctly classified 61.2% of cases. X^2^(21) = 74.257, *p* < .001. *CI* certainty interval, *SE* standard error. *N* = 779. *R*^2^ (Nagelkerke) = .121

### Faculty perceptions of academic dishonesty

Similarly, faculty overwhelmingly agreed that student academic honesty is important, with 80.0% agreeing strongly with this statement and 11.8% agreeing with this statement (Fig. 2b; Appendix D). Only 7.1 of faculty strongly disagreed, and 1.2% neither disagreed nor agreed. No faculty responded, “disagree” to this statement.

When it comes to whether or not faculty believe that incidents of academic dishonesty are increasing, faculty seemed split. The largest proportion of participants indicated that they neither disagree nor agree that incidents of academic dishonesty are increasing (51.8%), while 14.1% of faculty participants disagreed with this statement, 22.4% of participants agreed with this statement, and 11.8% strongly agreed (Appendix D).

### Do student witnessed incidents of academic dishonesty vary by gender, year of postsecondary experience, highest level of education, discipline, and/or perceptions?

**Fig. 3:**
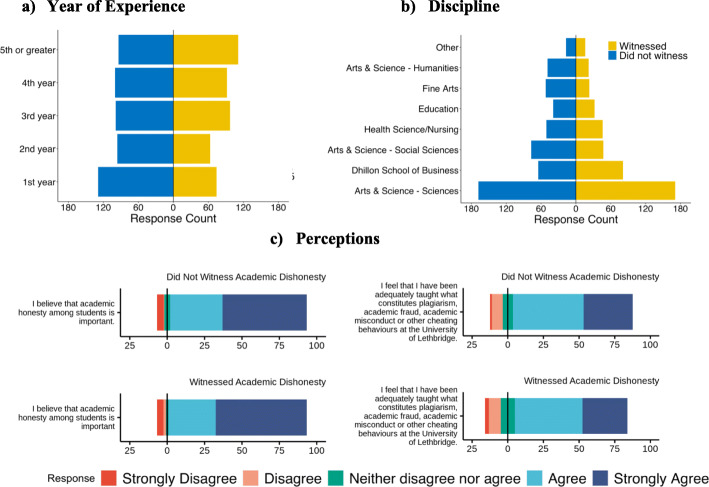
Student Witnessed Incidents of Academic Dishonesty by (**a**) Year of Experience, (**b**) Discipline, and (**c**) Perceptions

Our logistic regression analysis found no evidence that student reports of witnessing academic dishonesty varied by gender (*p* = .816; Table 4). We found witnessing varied by year of experience, discipline, educational attainment, and perceptions. In particular, we found that witnessing an incident of academic dishonesty increased by a factor of 1.38 (β = .321, *p* < .001; Fig. 3a) with each increasing year of experience, and that students within the disciplines of Arts and Science – Humanities, Arts and Science – Social Sciences, Education, and Fine Arts were all less likely to report witnessing an incident of academic dishonesty compared to students in Arts and Sciences – Sciences, while there was no effect for students in the Dhillon School of Business and Health Sciences/Nursing (Fig. 3b). Students who had a master’s degree were over 6 times more likely to report (β = 1.853, *p* = .021) witnessing an incident of academic dishonesty, but the error for this variable was quite large (SE = .801, CI = 1.327–30.656). We also found that compared to students who reported that they strongly believe that academic honesty among students is important, students who were indifferent in this perception (reporting that they neither disagreed nor agreed) were a third less likely (β = − 1.099, *p* = .030; Fig. 3c) to report that they had witnessed an incident of academic dishonesty. Finally, we found that compared to students who strongly agreed that they were adequately taught what constitutes constitutes plagiarism, academic fraud, academic misconduct or other cheating behaviours, students who were also indifferent in this perception were over two times more likely (β = .726, *p* = .021; Fig. 3d), and students who strongly disagreed were almost four times as likely (β = 1.305, *p* = .023; Fig. 3d), to report that they had witnessed an incident of academic dishonesty.

This regression classified 61.2% of cases correctly, and received an R^2^ (Nagelkerke) of .121, indicating that while our findings were significant, it was somewhat limited in the amount of variance in our data it can explain.

### Do faculty witnessed of incidents of academic dishonesty vary by gender, year of postsecondary teaching experience, and/or discipline?

**Fig. 4:**
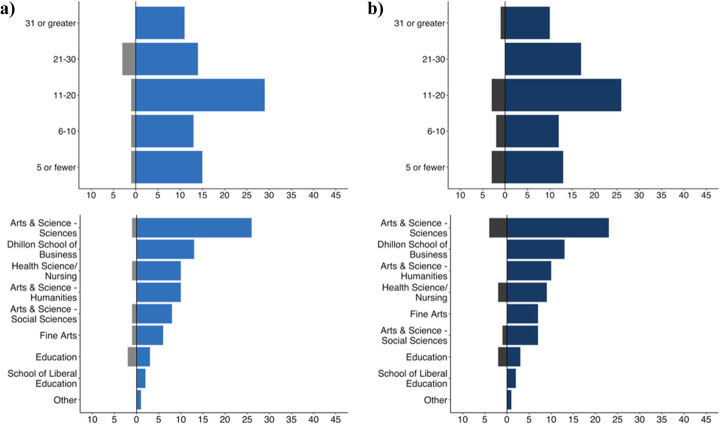
Faculty Reports of (**a**) Witnessing Incidents of and (**b**) Familiarity with University Policies towards Academic Dishonesty by Year of Experience, and Discipline

Overall, there was little variation in how often faculty witnessed incidents of academic dishonesty by gender, year of experience in postsecondary education, and discipline (Fig. 4a; Appendix E). All genders overwhelmingly (90.9%–100.0%) reported witnessing incidents of academic dishonesty. Witnessing incidents academic dishonesty across years of postsecondary experience was similar across years of experience, with 82.3%–100.0% of faculty participants reporting that they had witnessed some form of academic dishonesty while teaching, and 100% of participants surveyed who had 31 years or more teaching experience reported that they had witnessed academic dishonesty. Finally, the majority of disciplines reported that they had witnessed academic dishonesty in the past (the Education faculty were an exception, and were somewhat split in their responses, though this may reflect the small number of those surveyed).

### Does faculty familiarity with university academic dishonesty policies vary by gender, year of postsecondary teaching experience, and/or discipline?

The majority of faculty reported that they were familiar with university policies towards academic dishonesty across gender, year of experience in postsecondary education, and discipline teaching in (Fig. 4b; Appendix F). Similar to our findings to witnessing incidents of academic dishonesty, with the exception of education (40.0% “yes”, 60.0% “no”), all genders, years of experience in postsecondary education, and disciplines teaching in overwhelmingly reported (81.3%–100.0%) that they were familiar with university policies towards academic dishonesty. However, familiarity with university policies did appear to increase as years of teaching experience increased (the exception being the most experienced faculty, though this lower percentage may reflect the small number of faculty participants with 31 years or greater teaching experience compared to other experience categories).

## Limitations

Our study is limited in two main ways. Firstly, it was conducted within one university, making it difficult to generalize findings broadly beyond the questions for which we specifically asked, which concerned academic dishonesty at the University. Secondly, for many of our questions to students regarding their personal experiences with academic dishonesty, we did not specify rates (e.g., we only asked whether or not they had engaged in academic dishonesty, not how often they had done so). This might influence our interpretation, as we discuss below.

## Discussion

In general, our results show that both students and faculty overwhelmingly value academic integrity at our university. While these findings appear to be in contrast to earlier work pointing to much higher incidents of academic dishonesty (e.g., Christensen Hughes and McCabe 2006), many of the themes of student and faculty responses to our survey have been discussed across the academic literature and are linked to specific outcomes. We found that most students and faculty felt that academic integrity is important, and that they believed that they had been adequately taught university policies surrounding academic dishonesty. Consistent with our predictions, we found that whether or not students witnessed incidents of academic dishonesty varied by year of experience and discipline (though, this increase could also reflect an increase in opportunities to witness incidents of academic dishonesty). However, our predictions concerning whether or not students are familiar with university policies were only partially supported; while we did find that familiarity varied by year of study, we found no such variation by discipline. Furthermore, we found no evidence that faculty witnessing incidents of academic dishonesty, nor faculty familiarity with university policies surrounding academic dishonesty, consistently differed across gender, years of experience, or teaching discipline.

Taken together, our survey results suggest that the University of Lethbridge has not experienced a crisis with regard to contract cheating, a problem that appears to be growing across academic institutions in Canada and beyond (Lancaster 2019; Stoesz and Los 2019). This finding is consistent with data regarding incidents of academic dishonesty officially reported to the University. Coupled with our findings that the majority of students believe that academic dishonesty is important and were familiar with academic dishonesty university policies, our university’s low incidents of academic dishonesty corresponds with previous findings that students who cheat are also less likely to be familiar with institutional policy and who ascribe to social norms that do not discourage it (Jordan 2001; Murdock and Anderman 2006). These findings also suggest that probing students on their perceptions about academic dishonesty and university policy may be a useful indirect measure of academic dishonesty rates.

Additionally, in contrast to MacLeod and Eaton et al. (2020), our faculty does not overwhelmingly believe that incidents of academic dishonesty are increasing. Although there is no good evidence that cultural beliefs correlate with academic dishonesty, there is good evidence that the academic culture of an institution does (Bertram Gallant and Drinan 2008; McCabe 1993; McCabe et al. 2012; MacLeod and Eaton 2020) It is possible that this support for high quality teaching has had an impact at all levels for the work that takes place within the classroom, and this directed focus on teaching might function to support factors previously known to foster a culture of academic integrity. Although we did not probe student and faculty perceptions concerning the university’s academic culture, future research could determine whether or not these factors have influenced rates of academic dishonesty at our institution. It may well be that our university has a culture of academic integrity supported by our medium size and low student-faculty ratios; indeed, there is some evidence that university size can influence rates of academic dishonesty (Arnold et al. 2007; Bowers 1964; Davis et al. 1992). However, more research is needed, as a previous study of US undergraduates (and the only one we know of to consider student-faculty ratio) found no relationship between student-faculty ratio and student cheating behaviors (Tatum et al. 2018).

We also found a disconnect between the proportion of students who reported having previously engaged in academically dishonest practices (0.4–7.1%) and the proportion of students (45.0%) and faculty (92.0%) who reported witnessing others do so, as far fewer students reported engaging themselves than would be expected given the numbers witnessed by students and faculty. We believe there are a few possible reasons for this finding. The first is that multiple students could report witnessing the same academically dishonest incident, as we did not specify a frequency in our question. Alternatively, students might be underreporting their engagement in academic dishonesty or are engaging in some form not probed by our survey. Such a result would be consistent with the growing amount of evidence implicating a general lack of understanding as the primary motivation for a large amount of academically dishonest behaviors (Brimble 2016; Minarcik and Bridges 2015).

This kind of disconnect underscores the need for universities to develop institution-wide policies of academic integrity that are tailored not only to meet their unique needs, but that also contains certain “essential elements” previously found to describe effective policies (more below; c.f., Whitley and Keith-Spiegel 2001). While the evidence presented here reveals that incidents of academic dishonesty are low at our university, there were clear dissenters among our participants. A number of faculty surveyed reported that they were either indifferent to or strongly disagreeing with the importance of academic honesty, and the vast majority believed that incidents of academic dishonesty are increasing. We believe that if our university, and others with similar demographics, seeks to institutionalize academic integrity, there needs to a united effort to do so, one that focuses on three essential elements to academic polices identified by Whitley and Keith-Spiegel (2001). First, universities need a clear Definition/Explication of Prohibited Behavior. From our survey, we know that 21.8% of student participants reported that they were unfamiliar with what constitutes academic dishonesty at the university. This unfamiliarity might reflect a lack of a clear definition as to what constitutes prohibited behavior, given that currently, policies are spread across three domains. Clearly defining prohibited behavior includes providing diverse yet concrete examples of exactly what constitutes the spectrum of academically dishonest conduct, as the broad examples provided in general university policies are rarely effective (Whitley and Keith-Spiegel 2001). Secondly, universities should focus on Faculty Training. Though the majority of faculty participants in our study reported that they were familiar with what constitutes academic dishonesty at the university, 10.6% reported that they were unfamiliar, and this unfamiliarity appears to decrease with experience. It is likely that faculty become more familiar with experience, but explicitly training less-experienced faculty would remedy this issue faster. Finally, and most importantly, universities need to focus on Student Assistance/Orientation in order to ensure that students are directly informed of policies, and these policies are followed in the classroom. This could help to address student’s unfamiliarity with policies, as well as the 18.2% of student participants in our study who reported that they did not feel that they are adequately taught what constitutes plagiarism, academic fraud, academic misconduct or other cheating behaviors while in the classroom.

Finally, the novel coronavirus (COVID-19) pandemic has led to massive structural changes in every area of public life, as social distancing and quarantine measures have become commonplace (Odriozola-González et al. 2020). It is currently unknown to what extent this will affect academically dishonest practices. At our institution, we have seen a dramatic increase in reported instances of academic dishonesty, with a roughly 250% increase in reported cases between the Fall 2019 and Spring 2020 semesters (Appendix C). Similarly, we believe increases in cheating during online delivery has led to a number of stopgap solutions, such as the use of online proctoring services (Dimeo 2017). While the rapid switch from in-person to remote learning may have led to an increase in cheating, it also lets us reflect on the kinds of assessments that are employed in modern post-secondary education, including whether they reflect the needs of the current workforce (Evans et al. 2020).

## Future directions

Our study was conducted just before the emergence of SARS-CoV-2 and the subsequent shutdowns of in-person classes at the university on March 18, 2020, which resulted in the university transitioning to an exclusively online environment for the remainder of the Spring 2020 term. It is likely that the opinions expressed in the surveys described herein would vary given the move to exclusive online teaching environments because of a global pandemic. The transition to remote delivery in online environments has raised many concerns as to whether or not online environments will increase incidents of academic dishonesty (Harrison 2020; Mâță et al. 2020). Currently there is conflicting evidence as to whether or not online instruction increases rates of academic dishonesty (Harrison 2020) However, it is not known what kind of impact moving coursework initially designed for in-person instruction to remote delivery has on academic dishonesty, let alone established in-person university cultures post-covid (Evans et al. 2020). Given that the culture of an institution has been shown to impact rates of academic dishonesty (McCabe 1993; McCabe et al. 2012), future research should determine whether or not the current global pandemic has changed academic cultures, and if such cultural changes are reflected in academically dishonest practices.

## Data Availability

The datasets used and/or analyzed during the current study are available from the corresponding author (Dr. Oluwagbohunmi Awosoga) on reasonable request.

## Appendix A: List of Survey Questions Presented on the Student Survey

### Student Survey

1. Please identify your gender
  - Male
  - Female
  - non-binary
  - do not wish to identify
2. Please identify your current age
  - 17–19
  - 20–29
  - 30–39
  - 40–49
  - 50–59
  - 60–69
  - 70 or more
3. Please indicate your highest level of education
  - High school diploma
  - Diploma or Certificate
  - Bachelor’s degree
  - Master’s degree
  - Doctorate degree
  - Other (please list)
4. Please state your current year of post-secondary experience
  - 1st year
  - 2nd year
  - 3rd year
  - 4th year
  - 5 or greater
5. What discipline(s) are you currently enrolled in?
  - Arts & Science - Humanities
  - Arts & Science - Sciences
  - Arts & Science - Social Sciences
  - Dhillon School of Business
  - Education
  - Fine Arts
  - Health Science/Nursing
  - Other (please list)
6. In your own words, please define academic dishonesty.
7. Please indicate your level of agreement with the following statement:
  I believe that academic honesty among students is important.
  - Strongly Disagree
  - Disagree
  - Neither disagree nor agree
  - Agree
  - Strongly Agree
8. Have you personally witnessed any incidents of academic dishonesty in the past?
  - Yes
  - No
9. Please list up to five (5) examples of academic dishonesty that you have witnessed
10. Have you ever reused an assignment for another course (at the post-secondary level)
  - Yes
  - No
11. Have you ever turned in an assignment (at the post-secondary level) that someone else completed for you?
  - Yes
  - No
12. Have you ever turned in an assignment (at the post-secondary level) that you paid someone else to complete for you? (this does not include someone being paid to edit the paper for things like APA formatting, etc.)
  - Yes
  - No
13. Please indicate your level of agreement with the following statement:
  I feel that I have been adequately taught what constitutes plagiarism, academic fraud, academic misconduct or other cheating behaviours at the University
  - Strongly Disagree
  - Disagree
  - Neither disagree nor agree
  - Agree
  - Strongly Agree
14. Are you familiar with the policies at the university regarding academic dishonesty?
  - Yes
  - No
15. If you have any other comments that you would like to make about academic dishonesty on campus, please feel free to do so here. Please do not identify any specific individuals.
16. Upon the completion of this survey, you will have the opportunity to indicate your interest in participating in a follow-up focus group. Participation in a focus-group is completely voluntary.

## Appendix B: List of Survey Questions Presented on the Faculty Survey

### Faculty Survey

1. Please identify your gender:
  - Male
  - Female
  - non-binary
  - do not wish to identify
2. Please identify your current age:
  - 17–19
  - 20–29
  - 30–39
  - 40–49
  - 50–59
  - 60–69
  - 70 or more
3. Please indicate your highest level of education:
  - High school diploma
  - Diploma or Certificate
  - Bachelor’s degree
  - Master’s degree
  - Doctorate degree
  - Other (please list)
4. What is your current teaching position with the University?
  - Sessional Instructor
  - Term Appointment
  - Full-time Academic Assistant/Instructor
  - Assistant Professor
  - Associate Professor
  - Professor
  - Emeritus
5. Please state your current years of post-secondary teaching experience
  - 5 or fewer
  - 6–10
  - 11–20
  - 21–30
  - 31 or greater
6. What discipline(s) do you currently teach in?
  - Arts & Science - Humanities
  - Arts & Science - Sciences
  - Arts & Science - Social Sciences
  - Arts & Science - Social Sciences
  - Dhillon School of Business
  - Education
  - Fine Arts
  - Health Science/Nursing
  - Other
  - School of Liberal Education
7. In your opinion, what constitutes academic dishonesty?
8. Have you personally witnessed any incidents of student academic dishonesty in the past?
  - Yes
  - No
9. List some examples of academic dishonesty that you have observed. Please do not identify any specific individuals.
10. In your opinion, what constitutes plagiarism?
11. List some examples of plagiarism that you have observed. Please do not identify any specific individuals.
12. Please indicate your level of agreement with the following statement: “I believe that student academic honesty is important.”
  - ◦ Strongly Disagree
  - ◦ Disagree
  - ◦ Neither disagree nor agree
  - ◦ Agree
  - ◦ Strongly Agree
13. Have you reported academic dishonesty in the past?
  - Yes
  - No
14. On average, how frequently do you report?
  - < 1 per semester
  - 1 per semester
  - 2 per semester
  - 3 per semester
  - 4 per semester
  - 5 per semester
  - 6 per semester
  - 7 per semester
  - 8 per semester
  - 9 per semester
  - 10 per semester
  - > 10 per semester
15. How did you handle academic dishonesty in the past? (select all that apply)
  - Discussed with student
  - Reported to the Team Leader or Course Leader
  - Reported to the Chairperson
  - Reported to the Dean
  - Other (please list)
16. Please indicate your level of agreement with the following statement: “I believe in reporting incidents of academic dishonesty.”
  - Strongly Disagree
  - Disagree
  - Neither disagree nor agree
  - Agree
  - Strongly Agree
17. If you have witnessed incidents of academic dishonesty in the past, what are some of the reasons why you chose not to report them (choose your top 3) - Selected Choice
  - Does not apply: I always report
  - Lack of Chairperson or Dean Support
  - Unfamiliar with the academic misconduct institutional policies
  - Not intentional on the students’ part
  - Insufficient evidence
  - Reporting the incident was too much work
  - Lack of time
  - Potential for negative student evaluation(s)
  - Potential for negative peer evaluations(s)
  - Fear of verbal or physical assault
  - Opposed to confrontation
  - Potential for damaged relationships between the faculty and the student(s)
  - Potential for damaged relationships between the faculty member and their colleague(s)
  - Fear of negative impact from the administration personnel
  - Didn’t want to damage your reputation
  - Fear of losing your job
  - Other (please list)
18. Please indicate your level of agreement with the following statement: “It is important to communicate what constitutes academic dishonesty to my students.”
  - Strongly Disagree
  - Disagree
  - Neither disagree nor agree
  - Agree
  - Strongly Agree
19. I take the opportunity to communicate what constitutes plagiarism, academic fraud, academic misconduct and other unacceptable cheating behaviors to my students.
  - Never
  - Rarely
  - Sometimes
  - Frequently
  - Always
20. How satisfied are you with the support within the University towards reporting academic dishonesty?
  - Very dissatisfied
  - Dissatisfied
  - Neither dissatisfied nor satisfied
  - Satisfied
  - Very Satisfied
21. Are you familiar with the policies within the University towards reporting academic dishonesty?
  - Yes
  - No
22. How satisfied are you with the policies within the University towards reporting academic dishonesty?
  - Very Dissatisfied
  - Dissatisfied
  - Neither dissatisfied nor satisfied
  - Satisfied
  - Very Satisfied
23. I believe that incidents of academic dishonesty should be reported.
  - Never
  - Rarely
  - Sometimes
  - Frequently
  - Always
24. In my experience, I believe that incidents of academic dishonesty are increasing.
  - Strongly Disagree
  - Disagree
  - Neither disagree nor agree
  - Agree
  - Strongly Agree
25. How likely are you to report incidents of academic dishonesty in the future?
  - Very unlikely
  - Unlikely
  - Undecided
  - Likely
  - Very Likely
26. If you have any other comments that you would like to make about academic dishonesty on campus, please feel free to do so here. Please do not identify any specific individuals.

## Appendix C

**Table 5:** Student Academic Offenses Reported to University by Faculty

Semester	Academic Offenses (n)
Spring 2020	163
Fall 2019	46
Spring 2019	49
Fall 2018	37
Spring 2018	38
Fall 2017	29
Spring 2017	30
Fall 2016	34
Spring 2016	25
Fall 2015	36

## Appendix D

**Table 6:** Student and Faulty Perceptions Towards Academic Dishonesty

Statement	Response Options				
**Please indicate your level of agreement with the following statement: I believe that academic honesty among students is important.**	Strongly Agree (%)	Agree (%)	Neither disagree nor agree (%)	Disagree (%)	Strongly Disagree (%)
Students (*n* = 919)	537 (58.4%)	307 (33.4%)	27 (2.9%)	8 (0.9%)	40 (4.4%)
Faculty (*n* = 85)	68 (80.0%)	10 (11.8%)	1 (1.2%)	0 (0.0%)	6 (7.1%)
**Please indicate your level of agreement with the following statement: In my experience, I believe that incidents of academic dishonesty are increasing.**			Yes		No
Faculty (*n* = 85)			76 (89.4%)		9 (10.6%)

## Appendix E

**Table 7:** Whether Faculty Witnessed Incidents of Academic Dishonesty by Gender, Year of Experience in Postsecondary Education, and Discipline

Demographic (n)	Witnessed: Yes (%)	Witnessed: No (%)
**Gender**		
Do not wish to identify	1 (100.0%)	0 (0.0%)
Non-binary	1 (100.0%)	0 (0.0%)
Female	41 (95.3%)	2 (4.7%)
Male	40 (90.9%)	4 (%)
**Years of experience in postsecondary education**		
5 or fewer	15 (93.8%)	1 (6.2%)
6–10	13 (93.0%)	1 (7.0%)
11–20	29 (96.7%)	1 (3.3%)
21–30	14 (82.3%)	3 (17.8%)
31+	15 (100.0%)	0 (0.0%)
**Discipline teaching in**		
Arts & Sciences - Sciences	26 (96.3%)	1 (3.7%)
Dhillon School of Business	13 (100.0%)	0 (0.0%)
Arts & Science - Humanities	10 (90.9%)	1 (9.1%)
Health Science/Nursing	10 (100.0%)	0 (0.0%)
Fine Arts	8 (88.9%)	1 (11.1%)
Arts & Science - Social Sciences	6 (85.7%)	1 (14.3%)
Education	3 (60.0%)	2 (40.0%)
School of Liberal Education	2 (100.0%)	0 (0.0%)
Other	1 (100.0%)	0 (0.0%)

Percentages are representative of the total proportion of participants who indicated either “yes” or “no” to the question, “Have you personally witnessed any incidents of student academic dishonesty in the past?” Aggregated counts of each demographic category are also presented in Table 2

## Appendix F

**Table 8:** Faculty Familiarity with University Policies Towards Academic Dishonesty by Gender, Year, and Discipline

Demographic (n)	Familiar: Yes (%)	Familiar: No (%)
**Gender**		
Do not wish to identify	1 (100.0%)	0 (0.0%)
Non-binary	1 (100.0%)	0 (0.0%)
Female	39 (92.9%)	3 (%)
Male	38 (86.4%)	6 (%)
**Years of experience in postsecondary education**		
5 or fewer	13 (81.3%)	3 (18.7%)
6–10	12 (85.7%)	2 (14.3%)
11–20	26 (89.7%)	3 (10.3%)
21–30	17 (100.0%)	0 (0.0%)
31+	10 (90.9%)	1 (9.1%)
**Discipline teaching in**		
Arts & Sciences - Sciences	23 (85.2%)	4 (14.8%)
Dhillon School of Business	13 (100.0%)	0 (0.0%)
Arts & Science - Humanities	9 (81.8%)	2 (18.2%)
Health Science/Nursing	10 (100.0%)	0 (0.0%)
Fine Arts	7 (87.5%)	1 (12.5%)
Arts & Science - Social Sciences	7 (100.0%)	0 (0.0%)
Education	2 (40.0%)	3 (60.0%)
School of Liberal Education	2 (100.0%)	0 (0.0%)
Other	1 (100.0%)	0 (0.0%)

Percentages are representative of the total proportion of participants who indicated either “yes” or “no” to the question, “Are you familiar with the policies within the University towards reporting academic dishonesty?” Aggregated counts of each demographic category are also presented in Table 2

## Abbreviations

SE: Standard Error
SPSS: Statistical Package for Social Scientists
SARS-CoV-2: Severe acute respiratory syndrome coronavirus 2
